# Human induced pluripotent stem cell–derived liver-on-a-chip for studying drug metabolism: the challenge of the cytochrome P450 family

**DOI:** 10.3389/fphar.2023.1223108

**Published:** 2023-06-28

**Authors:** Isabel Tamargo-Rubio, Anna Bella Simpson, Joanne A. Hoogerland, Jingyuan Fu

**Affiliations:** ^1^ Department of Genetics, University of Groningen, University Medical Center Groningen, Groningen, Netherlands; ^2^ Department of Pediatrics, University of Groningen, University Medical Center Groningen, Groningen, Netherlands

**Keywords:** CYP450, hiPSC, OoC, drug metabolism, Organoids

## Abstract

The liver is the primary organ responsible for the detoxification and metabolism of drugs. To date, a lack of preclinical models that accurately emulate drug metabolism by the human liver presents a significant challenge in the drug development pipeline, particularly for predicting drug efficacy and toxicity. In recent years, emerging microfluidic-based organ-on-a-chip (OoC) technologies, combined with human induced pluripotent stem cell (hiPSC) technology, present a promising avenue for the complete recapitulation of human organ biology in a patient-specific manner. However, hiPSC-derived organoids and liver-on-a-chip models have so far failed to sufficiently express cytochrome P450 monooxygenase (CYP450) enzymes, the key enzymes involved in first-pass metabolism, which limits the effectiveness and translatability of these models in drug metabolism studies. This review explores the potential of innovative organoid and OoC technologies for studying drug metabolism and discusses their existing drawbacks, such as low expression of CYP450 genes. Finally, we postulate potential approaches for enhancing CYP450 expression in the hope of paving the way toward developing novel, fully representative liver drug-metabolism models.

## 1 Introduction

In drug research and development, it can take approximately 12–15 years before a drug can enter the market, with success rates as low as 10% for the progression of a compound from the preclinical to clinical phases of the pipeline (Pognan et al., 2023). To date, 2D and 3D preclinical models, including cultures from cell lines, primary cells or tissues (*in vitro*), computational models (*in silico*) and animal models (*in vivo*) are used to assess the safety and efficacy of new drug candidates (Mittal et al., 2019; Weaver et al., 2020; Wang et al., 2021; Smith, 2022). Animal models are frequently used to mimic relevant aspects of liver function and physiological responses to drugs prior to their entry into clinical trials. However, animal models do not recapitulate the human-specific drug response, and their implementation in drug testing raises ethical concerns regarding animal experimentation and welfare. In response, there has been a growing movement toward the use of human cell–based systems in drug testing.

In recent years, advances in organoid and organ-on-a-chip (OoC) systems have provided a unique opportunity to study organ complexity. Consequently, in 2022, the FDA passed a landmark bill allowing the use of OoC devices for drug-testing purposes (Rep. Buchanan, 2021). In light of this, several innovative OoC models have emerged, and specific OoC devices that mimic liver function and physiology have been developed for the purposes of drug metabolism and toxicity studies. The development of liver-on-a-chip models utilizing primary hepatocytes, the current ‘gold standard’, has been extensively explored (Apte & Krishnamurthy, 2011; Docci et al., 2022; Ewart et al., 2022). However, given the limited availability of primary human hepatocytes, the use of human induced pluripotent stem cells (hiPSCs) has been a subject of growing interest (Kaur et al., 2023). The advantage of hiPSC-based models is that these cells can be non-invasively obtained from patient material and differentiated into a plethora of cell types, including hepatocytes. Additionally, the use of hiPSCs brings with it the capacity for patient-specificity as they exhibit and retain the genetic background of the donor. However, to date, the reduced expression of cytochrome P450 genes (CYP450), key regulators in drug metabolism, in hiPSC-derived hepatocytes limits their application in drug studies.

In this review, we provide an overview of the human-based cellular models currently used in drug testing and toxicity studies and discuss the potentials and challenges of emerging liver-on-a-chip models. In particular, we focus on specific challenges related to insufficient expression of CYP450 genes in hiPSC-based liver-on-a-chip models and we postulate potential approaches to improve OoC systems for drug-metabolism studies and toxicity analysis.

## 2 *In vitro* 2D model systems

Current human-based 2D cellular model systems for drug testing include primary hepatocytes, cancer cell lines (e.g., HepG2), liver tissue slices and hepatocytes differentiated from either adult multipotent stem cells (ASC) or hiPSCs (Zeilinger et al., 2016; Roselló-Catafau et al., 2022). Primary hepatocyte models are advantageous because they show hepatic gene expression and carry the genetic background of the donor, but they also have several limitations. Firstly, isolation of primary hepatocytes is challenging as they are obtained from patient liver biopsies and their maintenance *in vitro* requires complex protocols. Secondly, their acquisition from healthy donors is infrequent due to the invasive nature of biopsy retrieval (Kaur et al., 2023). Therefore, findings generated from models based on primary hepatocytes are often subject to the influences of the disease carried by the donor, thereby impacting translatability of these findings to the non-diseased population (Green et al., 2017). Thirdly, primary hepatocytes can only survive up to a week in 2D culture, which complicates their utility for long-term drug studies (LeCluyse, 2001; Kaur et al., 2023).

Liver-specific cancer cell lines, such as HepG2, can be maintained in culture over longer periods of time (Arzumanian et al., 2021), and they can be easily cultured, expanded and genetically modified. However, these cell lines often carry oncogenic DNA alterations that make them less ideal for replicating an individual’s genetic background. In addition, HepG2 cells have genetic abnormalities that are not present in primary hepatocytes, and they fail to accurately recapitulate many liver-specific functions such as metabolic capacity and comparative drug uptake (Wilkening et al., 2003; Wiśniewski et al., 2016; Arzumanian et al., 2021). Together, these factors reduce their applicability and predictive value in drug-testing models.

Precision cut liver slices (PCLS) have recently arisen as a promising alternative liver model, as they retain the complex physiological architecture of the native liver, including its specific cellular composition and organization. Although PCLS can provide a more realistic model of liver metabolism than traditional cell culture models, they lack the physiological cues present *in vivo*, such as blood flow, which limits their relevance for studying *in vivo* drug metabolism. Moreover, similar to primary hepatocytes, PCLS have limited availability, large variability and a short life-span for drug testing (Olinga & Schuppan, 2013; Biel et al., 2022).

Differentiated tissues derived from ASCs and hiPSCs are valuable alternatives to cancer or immortalized liver cell lines as they carry the same genetic makeup as the donor. As a consequence, models derived from stem cells are attractive for drug-testing purposes because they also make it possible to gain insight into individual-specific drug response and thus support development of personalized medicine (Lynch et al., 2019; Xie et al., 2021)*.* Another advantage of the use of stem cells is the possibility to generate any cell type present in the tissue of interest, allowing the generation of more complex cultures involving more than 1 cell type. The main difference between ASCs and hiPSCs is in their epigenetic profile, which is defined intrinsically by the original tissue source. While ASCs retain their original epigenetic markers due to their isolation from mature tissue, hiPSCs lose their epigenetic memory during the stringent reprogramming protocols necessary to induce pluripotency (Polo et al., 2012). hiPSCs thus exhibit an ‘embryological’ phenotype compared to the ‘mature phenotype’ of ASCs (Kim et al., 2010; Goversen et al., 2018). Next to the difference in epigenetic markers, ASCs and hiPSCs differ in the methods required for their isolation. Whereas ASCs are isolated directly from the tissue of interest, hiPSCs are obtained in a non-invasive manner from different adult tissue cell sources such as erythroblasts isolated from the PBMC fraction of blood or from urine-derived epithelial cells. Consequently, the increased utility and availability of hiPSCs have directed research toward the development of numerous protocols that outline culturing methods to obtain hepatocyte-like cells (HLCs) for drug metabolism studies (Mallanna & Duncan, 2013; Mun et al., 2019; Pettinato et al., 2019; Thompson & Takebe, 2020; Kim et al., 2022; Li et al., 2022; Messina et al., 2022; Pettinato, 2022; Weng et al., 2023; Zhang et al., 2023). However, it should be noted that although 2D cultured hiPSCs have advantages compared to cancer cell lines and primary hepatocytes in terms of isolation and culturing, they still do not fully represent the structural and physiological complexity of the human liver. Model development is therefore now being focused on 3D systems that can incorporate multiple cell types, thereby allowing accurate representation of the complex cellular interactions found within the native liver.

## 3 Liver-on-a-chip: recapitulating tissue complexity

The liver is a highly structured organ that supports its central role in nutrient and drug metabolism. Hepatocytes account for ∼70% of the total liver content, while the remaining liver cells consist of non-parenchymal cells, such as cholangiocytes, liver sinusoidal endothelial cells (LSECs), hepatic stellate cells (HSCs) and immune cells. Hepatocytes are organized in hexagonal subunits called lobules, which can be further divided into the periportal zone surrounding the portal triads (portal vein, hepatic artery and the bile duct), the pericentral zone adjacent to the central vein and the midlobular zone in between. Nutrient and oxygen-rich blood flows from the gut via the portal vein to the liver, passes through a network of liver sinusoids and leaves the parenchyma via the central vein. Hepatocytes take up and secrete nutrients and sense hormones, creating a nutrient and oxygen gradient over the different zones, as extensively reviewed by others (Ben-Moshe & Itzkovitz, 2019; Paris & Henderson, 2022). Moreover, hepatocytes are polarized (Gissen & Arias, 2015), forming a cell layer that separates the sinusoidal blood flow and its counter-current canalicular bile flow.

An emerging 3D model system in current development is the microfluidic OoC device. The use of OoC devices allows precise emulation of specific aspects of organ characteristics known to impact cell behavior and, in turn, metabolism. The most evident feature that OoC systems introduce is the presence of two or more channels. In some OoC platforms, these channels can be independently controlled, generating a unique microenvironment in media and cell-type composition. The design and complexity of the OoC device can be adjusted depending on the needs of the researchers, going from the most simplistic two-lane architecture separated by a porous membrane (Ewart et al., 2022) to more complex designs that try to recapitulate the exact liver lobule microarchitecture and oxygen gradients (Du et al., 2021). All the different cell types present in the liver can be potentially cultured in a single OoC device, allowing better recapitulation of the tissue complexity. OoC not only provides the physical space to culture all these cell types together in an appropriate configuration but also other features like mechanical forces, shear stress, and media detoxification driven by the flow, the most important feature of these devices (Dalsbecker et al., 2022). In terms of flow, we also find interesting differences between devices, from platforms in which the media is recirculated (Docci et al., 2022), mimicking the blood circulation in the body, to others in which the media follows a unidirectional flow, entering and leaving the device at a specific speed, which better mimics the detoxification process and makes sampling easier. Importantly, two-lane chips can be customized to recapitulate the counter-current flow of blood and bile.

To date, liver-on-a-chip models mainly incorporate primary hepatocytes, which can be co-cultured with non-parenchymal cell types such as LSECs, Kupffer cells (KCs) and HSCs (Ewart et al., 2022; Kato et al., 2022). Importantly, OoC devices have resolved several limitations characteristic of 2D primary hepatocyte cultures. Namely, liver-on-a-chip models enhance primary hepatocyte viability, increasing their life-span in culture. Additionally, the presence of multiple channels enables the incorporation of liver immune cells, enabling deeper insights into the effect of these cells by mimicking their interactions *in vitro*. The value of a liver-on-a-chip model that relies on primary cells has been demonstrated by studies showing its ability to correctly predict the toxicity of 27 different drugs with known hepatotoxic or non-toxic behavior (Ewart et al., 2022), including human-specific drug toxicity (Jang et al., 2019).

Though OoC based on primary hepatocytes has proven to be a promising resource for accurate drug toxicity prediction, these models still face issues regarding the limited availability of primary hepatocytes and the often diseased nature of these cells due to the donor-specific phenotype. In light of this, it has been suggested that the use of hiPSC-derived HLCs in OoC devices could help overcome these limitations, providing a novel and personalized liver-on-a-chip model for drug testing. The formation of HLCs from hiPSCs has been extensively characterized, and HLCs have been incorporated into multiple model systems for physiological and disease modeling of the liver (Bircsak et al., 2021; Hendriks et al., 2023). The current differentiation protocols involve a series of stages in which the hiPSCs are first differentiated to the definitive endoderm, the germ layer from which the liver is generated, and subsequently formed into HLCs. Full differentiation can be completed in 19–25 days depending on the growth factor composition present in the media at each stage and the protocol used (Mallanna & Duncan, 2013; Mun et al., 2019; Pettinato et al., 2019; Kim et al., 2022; Pettinato, 2022). Differences in protocols can significantly affect and alter gene expression in HLCs. Therefore, as HLCs are often used for different purposes and thus require different hepatocyte-specific phenotypes and maturation statuses, there is little consensus on optimal protocol use. Although HLCs express common hepatocyte markers such as HNF4ɑ, ALB and CK18, they are often limited in their metabolic capacity. Specifically, they show reduced expression of a group of key metabolic enzymes known as the cytochrome P450 family (CYP450).

## 4 CYP450 enzymes are key regulators in drug metabolism

The CYP450 family comprises 57 putatively functional genes, grouped into 18 families and 44 subfamilies, that encode an array of enzymes that function as ubiquitous catalysts. Among these, the most common enzymes are CYP3A4/5, CYP2C9, CYP2D6 and CYP2C19, which are responsible for 80% of drug oxidation in the adult liver (Ahmed et al., 2018). During Phase I metabolism, liver-specific CYP450s modulate drug compounds via sequential oxidation-reduction reactions. This includes formation of radical intermediates that are necessary for the further processing in Phase II metabolism, which functions to inactivate and increase the polarity of the metabolite formed, facilitating its excretion from the body (Zhou et al., 2009). The complexity of CYP-mediated drug biotransformation arises from three features: the broad substrate-specificity of a single CYP450 isoenzyme, the multiplicity of enzymes involved in a single drug’s biotransformation and the formation of various metabolites via different reactions. An example of this three-fold complexity is the transformation of racemic warfarin, where at least seven different hydroxylated metabolites are produced, depending on the activity of specific CYP450 isoforms (Zhao et al., 2021). Thus, the hepatic expression and functionality of CYP450s are vital determinants of drug turnover rate, efficacy and toxicity.

CYP450 genes are often polymorphically expressed, and allelic variants can produce enzymes with increased, ablated or reduced activity. Genetic variants such as copy number variations and single nucleotide polymorphisms are the most established factors that cause differential expression and functional changes within the core of the enzyme, leading to distinct metabolic activity. CYP450 phenotypic populations can be categorized as ultra-rapid, rapid or slow metabolizers, with each phenotypic status having specific implications for drug efficacy and toxicity. Depending on the phenotype, drug-mediated toxicity or effectiveness can be anticipated in a dose-dependent manner. For instance, poor metabolizers are susceptible to toxicity due to toxic accumulation (Zhao et al., 2021), whereas rapid metabolizers may experience severe clinical outcomes if the polymorphism is harbored in isoforms that form toxic intermediates or present with lower efficacy of the treatment because the drug is broken down into metabolites and excreted before it can reach the minimum effective drug concentration in plasma. These pharmacogenetic influences on CYP450 activity and expression can produce significant variations in drug efficacy and, in some instances, contribute to drug attrition from the market (Zhao et al., 2021). Investigating the effect of CYP450 polymorphisms in preclinical drug metabolism models enables an increased understanding of their clinical implications for toxic effects, enabling personalized adjustments of dosage regimes.

Overall, variation in treatment response is mainly attributed to the different genetic profiles of each individual and has led to the establishment of a new field of study known as pharmacogenetics (Luzum et al., 2021). To fully recapitulate and understand the impact of these polymorphisms, hiPSC-based OoC devices represent a promising method for personalized drug metabolism studies. In combination with their ability to incorporate patient-specific genetics, the increased tissue complexity in these models can help recapitulate the functional consequences of CYP450 polymorphisms. However, as previously discussed, insufficient CYP450 expression is still a roadblock to the success of these models. The use of CYP450 inducers, such as rifampicin, proves challenging for the study of patient-specific metabolic profiles. Artificially increasing the expression and functionality of CYP450 enzymes leads to an increased likelihood of drug-drug interactions with the compound of interest and alters the patient-specific phenotype (Chen & Raymond, 2006; Hakkola et al., 2020; Ramsden & Fullenwider, 2022). Therefore, a vital next step for the increased predictive success of liver OoC devices is the enhancement of CYPs within hiPSC-derived HLCs, thereby preserving true CYP450 expression.

## 5 Improving CYP450 expression in hiPSC-based liver-on-a-chip

**FIGURE 1:**
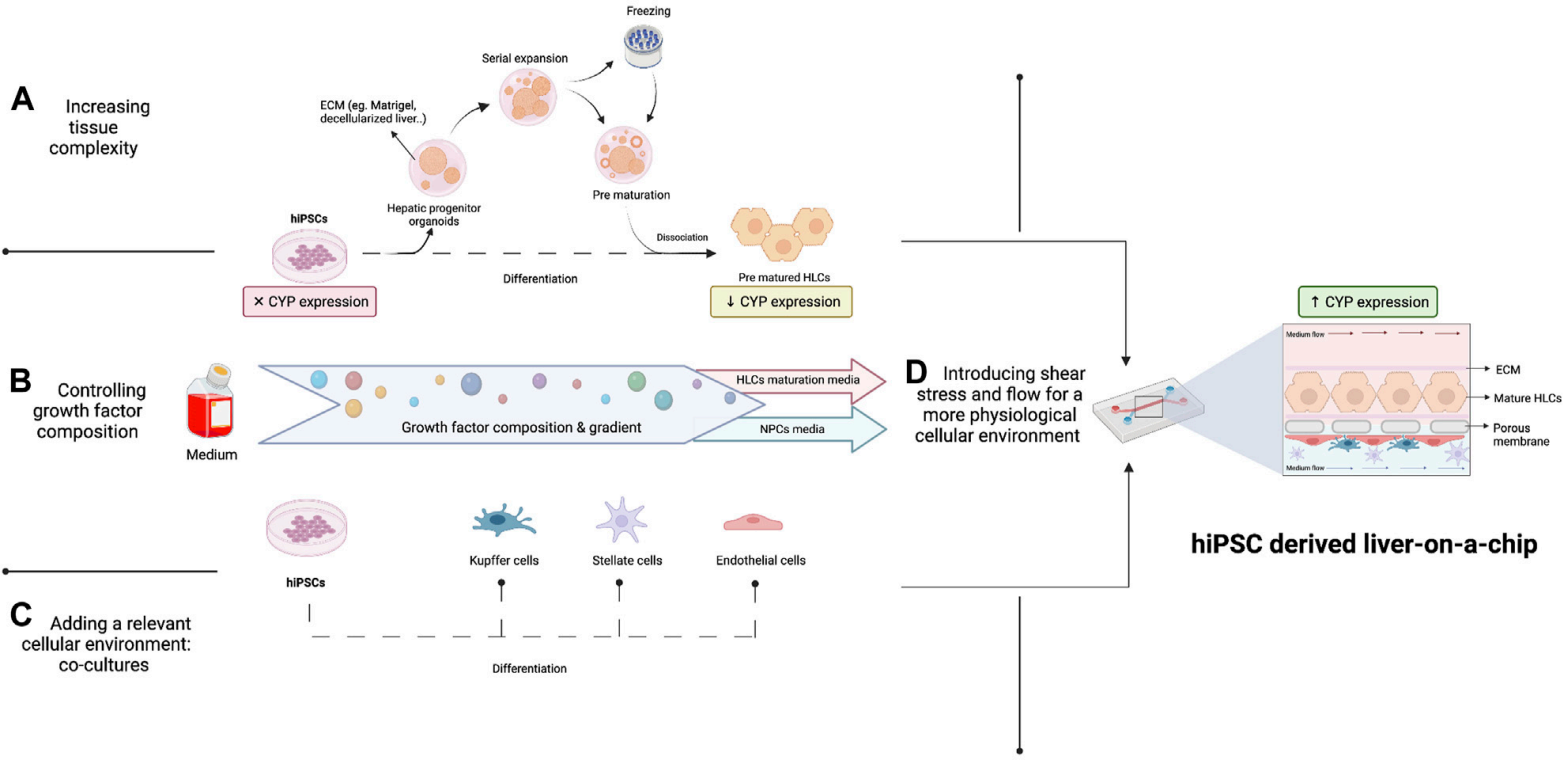
Improving CYP450 expression in a hiPSC-derived liver-on-a-chip. Enhanced CYP450 expression by HLCs could be achieved in three ways: **(A)** by increasing tissue complexity by the formation of organoids in the optimal supportive matrix and subsequent prematuration, **(B)** by controlling the composition and concentration of different growth factors in cell medium during hiPSC differentiation and **(C)** by differentiating hiPSCs in a relevant cellular environment by adding other liver cell types, which will increase CYP450 expression in HLCs. **(D)** In addition, physiological cues such as shear stress and flow will positively affect CYP450 expression by hiPSCs on-chip by better mimicking liver physiology.

To date, classic 2D culture and differentiation protocols are insufficient to provide the proper environment for hiPSCs to differentiate and fully mature. The main feature that needs to be recapitulated in order to use OoC systems for personalized medicine is a CYP450 expression in HLCs that is comparable to the donor’s original liver phenotype. As shown in Figure 1, several methodologies have been adopted to address this issue, including alteration of growth factors during hiPSC differentiation, co-culture of different cell types, CYP450 expression inducers and 3D organoid culture.

### 5.1 Growth factors and the ECM in the differentiation process

The protocols for differentiating hiPSCs into hepatocytes have multiple stages that mimic fundamental endogenous developmental pathways. Commonly used differentiation protocols include growth factors such as Activin A, Wnt3 or CHIR99021 for induction of the definitive endoderm, followed by FGF2 and BMP4 for hepatic progenitor differentiation and HGF and Oncostatin M for the hepatocyte maturation stage (Mallanna & Duncan, 2013; Mun et al., 2019; Overeem et al., 2019; Pettinato et al., 2019; Thompson & Takebe, 2020; Kim et al., 2022; Pettinato, 2022). Kim et al. demonstrated that withdrawal of specific growth factors previously thought essential for proper hepatocyte differentiation, such as R-spondin 1, EGF and Noggin, surprisingly did not affect hepatocyte differentiation but did result in a significant increase in CYP450 expression (Kim et al., 2022). Another parameter known to impact the HLC differentiation process is the ECM. In standard practice, hiPSCs are cultured on Matrigel^®^ coated plates or between two layers of Matrigel^®^ (“sandwich-based approach”) to enhance their attachment and facilitate their polarization during the differentiation process (Overeem et al., 2019; Li et al., 2022). Interestingly, alternative culturing matrices such as ECM composed of decellularized rat liver tissue have been explored to augment HLC maturation in the hope of increasing CYP450 expression. When compared to the Matrigel®-based sandwich approach, six tissues showed increased CYP450 expression in HLCs due to their specific ability to correctly mimic the real physiological microenvironment of the liver (Agmon & Christman, 2016; Wang et al., 2016; Robertson et al., 2018; Parmaksiz et al., 2020; Acun et al., 2022). These results suggest that, overall, further improvement and fine-tuning of the culturing environment can support hepatocyte maturation and enhance CYP450 expression.

### 5.2 Co-cultures to improve cell maturation

Multiple studies have highlighted the beneficial effects of co-cultures for improving the maturation status of hiPSC-derived HLCs, suggesting that complex cell-cell interactions may be required to induce optimal metabolic activity following differentiation (Jaramillo & Banerjee, 2012; Lee et al., 2018; Kovina et al., 2021). Models that incorporate non-parenchymal cells such as LSECs, KCs or HSCs, alongside the hepatocytes enable realistic recapitulation and elucidation of cell-cell interactions. Developing model systems that include the diverse heterogeneity of liver cell types is paramount to accurately recapitulate the complex cell-cell and cell-matrix interactions known to contribute to hepatocyte maturity. Furthermore, Pettinato et al. showed that the addition of human adipose microvascular endothelial cells during hiPSC differentiation led to a more mature cell phenotype, as determined by *HNF4ɑ* and *CK18* expression, CYP450 activity and albumin secretion (Pettinato et al., 2019). Multiple cell types thereby enhance CYP450 expression; however, like all 2D models, the ability to recapitulate organ complexity remains limited. Therefore, the formation of organoids, small representations of an organ, may prove a promising route for generating an increased level of cellular interaction and 3D orientation and complexity.

### 5.3 3D organoid configuration

Via mimicry of organogenesis, 3D liver tissue can be engineered using hiPSCs, culturing HLCs alone or in co-culture in an organoid or spheroid configuration. Importantly, these structures can synthesize and secrete serum proteins (Messina et al., 2022), and they can exhibit detoxification (Kim et al., 2022). Most importantly, the formation of a 3D configuration has been directly linked to increased CYP450 expression.

hiPSC-derived hepatic organoids and primary human hepatocytes have shown comparable sensitivity to compounds that induce liver injury (Zhang et al., 2023). Moreover, hiPSC-derived hepatic organoids show higher expression and increased activity of CYP450 compared to 2D HLCs and HepG2 (Weng et al., 2023). One of the potential reasons for the diminished expression of CYP450 genes in 2D cultures was described by Kim et al. when studying CYP450 DNA methylation patterns and cellular configuration in hiPSC-derived HLCs cultured in 2D and in an organoid configuration (Kim et al., 2022). They concluded that the reduced CYP450 expression observed in 2D systems could be partially explained by increased DNA methylation of specific CYP450 promoter regions. It has also been described that the addition of LSECs to HLC-organoids improved the expression of several CYP450 enzymes, including CYP3A4, by at least 1.5-fold (Ardalani et al., 2019), suggesting that cell-cell interactions may enhance metabolic capacity and highlighting the importance of paracrine signaling (Park et al., 2015; Kim et al., 2022). One major disadvantage of organoid cultures is the tedious culture methods and the challenge of controlling the growth and size of these 3D structures, which increases the variability between biological replicates (Harrison et al., 2021)

These examples highlight the importance of the 3D environment for the differentiation and maturation of HLCs. The integration of liver organoids containing multiple cell types with OoC devices would merge the advantages of both systems, namely, improved maturation of HLCs, capacity for co-cultures and enhanced HLC metabolic capacity. In addition, the inability to control growth and have comparable biological replicates would disappear because the organoids would be dissociated into single cells before seeding onto the OoC. The potential of this integrated system was explored by Zhang et al., who generated hepatic liver organoids with multiple cell types. These hepatic organoids showed positive immunofluorescence results for hepatocytes (*HNF4α*), HSCs (*α-SMA*) and KCs (*CD68*), and those cells also presented improved metabolic capacity and maturation profiles than HLCs cultured in 2D (Zhang et al., 2023). Combining these organoids with OoC increased secretion of albumin by 2–3-fold and expression of CYP450 enzymes by 3–5-fold compared to hepatic organoids cultured in static conditions, demonstrating the advantages of microfluidics.

## 6 Conclusion and future perspectives

In the clinic, the adoption of a ‘one-drug-fits-all’ policy has been scrutinized due to its inability to provide patients with universally effective treatments. This has driven a growing demand for the development of personalized models that incorporate an individual’s genetic makeup, a factor known to impact drug metabolism and toxicity. Alongside the inability to model interpersonal differences in patient drug response, the current model also has direct negative implications for the pharmaceutical industry, increasing the length of time it takes for a drug to enter the market due to unforeseen side effects. One current bottleneck is the availability of good preclinical models that can accurately recapitulate human physiology and the genetic differences between patients. To solve this issue the development of an ideal preclinical model for personalized drug testing is required and several criteria must be met. Firstly, it should have the capacity to directly emulate the physiology and functionality of the organ system under study. Secondly, it should have increased translatability to the human system; therefore, as opposed to being based on animal models, it ought to be human cell–based. Finally, it should be able to incorporate an individual’s genetic background.

**FIGURE 2:**
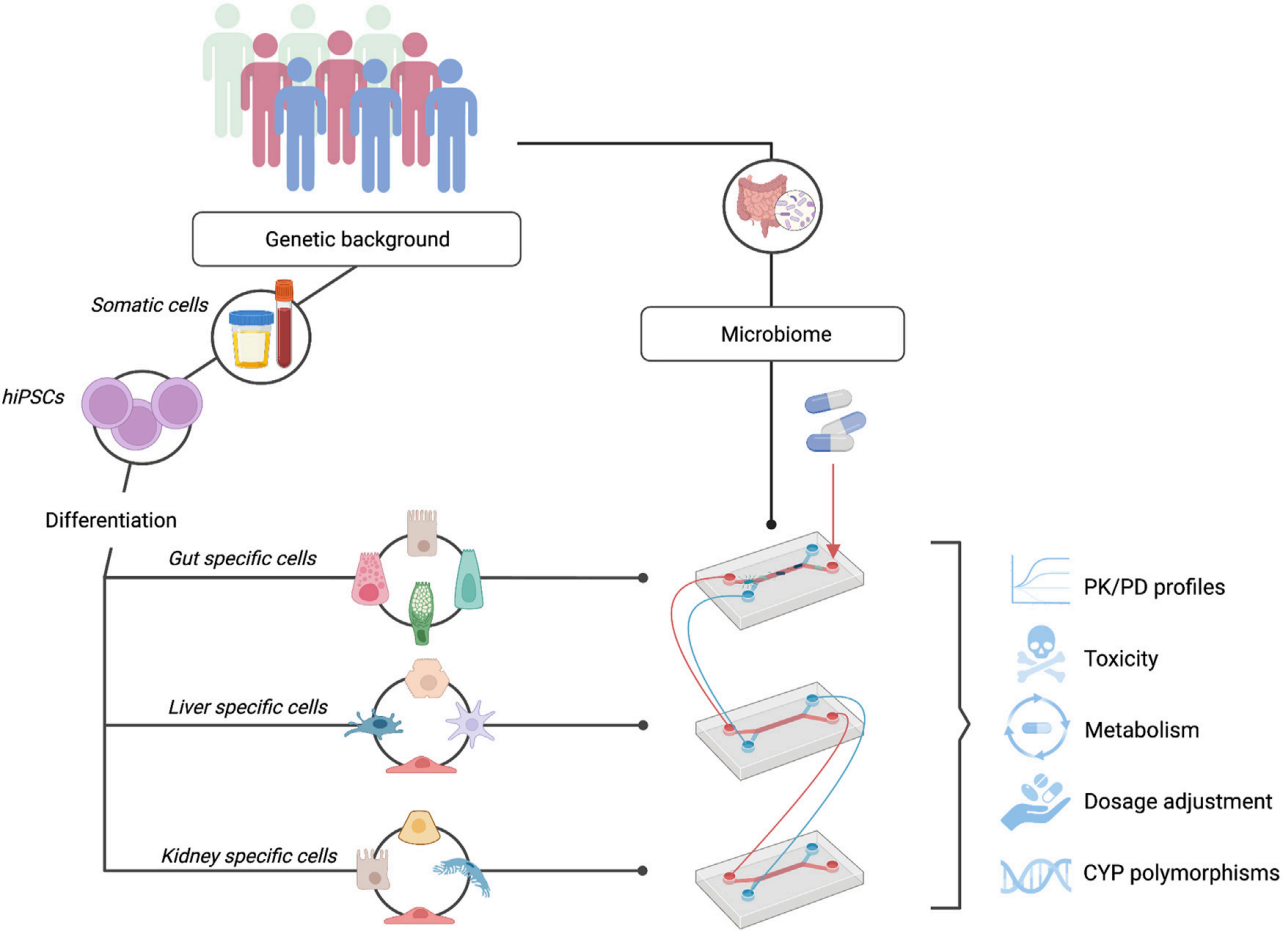
hiPSC-OoC Systems: A Promising Model for Personalized Drug Testing. hiPSCs can be differentiated into the cells of interest while maintaining donor-specific phenotypes. Interconnecting multiple organs-on-chip such as the gut, liver, and kidney, allow the study of personalized pharmacokinetic and pharmacodynamic (PK/PD) profiles. In combination with individuals’ microbiome on-chip, a fully representative and personalized system can be obtained to study drug toxicity and metabolism, determine personalized drug dosages, and investigate the effect of patient-specific CYP450 polymorphisms.

In this review, we propose that the combination of OoC systems with hiPSCs represents a promising preclinical model for personalized drug testing (see Figure 2). Although the ideal model would be a full liver generated *in vitro* via genetic and tissue engineering, our current knowledge and technology is limited. However, the development and use of hiPSC-derived OoC systems represents a unique opportunity to innovate current preclinical drug-testing models, allowing us to modify specific cues that are known to alter the process of drug metabolism. Specifically, due to the importance of CYP450 gene expression in drug metabolism, the capacity of these models to allow the modulation and integration of these genes is a crucial step forward. Currently, lack of or diminished expression of CYP450 genes in HLCs represents the major challenge in the development and use of hiPSC-derived liver-on-a-chip systems in personalized medicine. In this review, we have extensively discussed different approaches that could enhance CYP450 expression. In summary, we conclude that accurate recapitulation of various aspects specific to the native liver, such as the presence of liver-specific cell types, 3D configuration and the addition of specific growth factors at the right time points, can directly impact the ability of these cells to mature and express CYP450s. The possibility to represent donor-specific genotypes, and thus their phenotypic expression of CYP450s, can be argued as one of the most important variables required for the development of effective preclinical models. The importance of patient-specific polymorphisms in these enzymes has already been proven, and this genotype information is slowly being integrated into the clinic in the form of pharmacogenetic passports, an approach that has been shown to lead to a 30% reduction in adverse drug reactions (Bangma et al., 2020; Swen et al., 2023). The development of representative models will allow us to answer specific research questions and inquiries about the impact of genetics on drug metabolism, streamline and accelerate the drug development pipeline and help patients and clinicians appropriately adjust dosages.

Although OoC devices present an exciting avenue for future research, these devices can only partially capture the complex mechanisms occurring within an organ system. Liver zonation, hepatocyte polarization, or the spatial distribution of cells in the liver lobules are also key for the recapitulation of specific features. The hepatocytes distributed along the pericentral and periportal vein differ in the expression of several markers and, notably, drug metabolism genes are mainly expressed in the pericentral zone (Brosch et al., 2018; Sanchez-Quant et al., 2022). OoC systems with a focus on drug metabolism that fully recapitulate zonation are currently lacking, although attempts to address this are ongoing (Tonon et al., 2019; Danoy et al., 2020).

Complexity should also not be seen as a one-organ feature. Inter-organ communication plays an important role in the context of pharmacokinetics and pharmacodynamics (PK/PD) and human drug metabolism is not exclusive to the liver’s metabolic capacity. For example, it is well-established that enterocytes present in the intestine can modulate drug absorption and metabolism (Ho et al., 2017) and the kidney is responsible for the excretion of these compounds. In recent years, the complex interaction of the gut microbiome in drug response has also been an emerging research avenue. Development of fully representative personalized drug-testing models will require incorporation of the interplay along the microbiome-gut-liver axis (Pant et al., 2022; Spanogiannopoulos et al., 2022). Complex modeling using hiPSCs to generate cell types specific for the intestine and the liver will allow an individual’s genetics and gut microbiome to be taken into account simultaneously in drug testing. Altogether, OoC and stem cell technologies are co-evolving to generate valuable *in vitro* systems that will ultimately directly impact patients’ health, moving toward personalized medicine.

## Abbreviations

OoC, organ-on-a-chip; hiPSC, human induced pluripotent stem cells; HLCs, hepatocyte-like cells; CYP450, cytochrome P450; PCLS, precision cut liver slices; NPCs, non-parenchymal cells; KCs, Kupffer cells; LSECs, liver sinusoidal endothelial cells; HSCs, human stellate cells; PK/PD, pharmacokinetics/pharmacodynamics.
